# Supplementary material for: Towards an objective theory of subjective liking: A first step in understanding the sense of beauty
Source: PLoS One. 2023 Jun 23;18(6):e0287513. doi: 10.1371/journal.pone.0287513 (PMC10289447; doi:10.1371/journal.pone.0287513)
Supplement: Attachment — Submitted filename: answersV1.pdf [file pone.0287513.s002.pdf]

Towards Measuring the Sense of Beauty
answers to the reviews (**note**: the title has changed in the current version)

S. Mazzacane[1], M. Coccagna[1], F. Manzella[2], G. Pagliarini[2], A.V. Sironi[3], A. Gatti[4], E. Caselli[1], G. Sciavicco[2]

To the Managing Editor

Dear managing editor, thank you for taking the time to handle our paper. We feel that the reviewers appreciated the paper, and that we were able to answer all their concerns. With respect to the editor's questions:

1. **The Authors have to carefully specify how the present manuscript differs from this previous publication: [. . .].** First, the conference paper was based on a prototypical version of our software/methdology, which, in turn, was not explained in detail; in this paper the method is carefully explained, to ensure reproducibility. Second, the dataset in the conference paper was given in a single version; in this paper, there are three versions of datasets, which contribute to a better understanding and validation of the results. Third, the experiment parameters were arbitratrily fixed in the conference paper, and systematically explored in the journal version. Fourth, the conference paper did not contain the learned models, which are on the contrary reported in this one. Summarizing, whoever reads the conference version cannot deduce, if not superficially, the content of the journal paper. Having said that, we recognize an overlap in the text, which we have now fixed, in particular, as we explain below, in the related work and assessment of the results.

2. **The Authors should state how they selected their sample size, with appropriate explanations, and possibly adding a power analysis, if they performed one.** One essential factor is that machine learning techniques differ from inferential statistics ones, specifically in terms of necessary hypothesis. Machine learning techniques are specifically designed not to require any prior hypothesis on the distribution(s); therefore, it would make no sense to perform power analysis or apply similar hypothesis-testing methods. By choosing to approach this problem with ML, we promote the principle that 'techniques should adapt to data, not data to the techniques'. In other words, ML does not require minimum size, or normally distributed data, because it is designed to adapt to the case at hand. This particular experiment was not designed to the purposes of this study only, and the price to be paid for having 'natural conditions' includes less freedom in choosing the size and other parameters w.r.t. the subjects. We added some text to explain this point in the paper. Observe, on a related note, that datasets usually employed for these kind of researches are generally smaller than ours (except the public ones).

3. **Because of Plos One policy, Authors are required to make their data publicy available.** We did not realize that the policy was strict. We apologize for the misunderstanding on our side, and we are willing to disclose the necessary data as per Plos One rules.

4. **The Authors should expand their discussion section, trying to further discuss their results also highlithing shared aspects and possible discrepancies with previous neuroaesthetic evidence. Also it seems to me that Authors' review of previous EEG literature on neuroaesthetic is a bit scarce. Finally I fully agree with Reviewer 2 when they expressed the importance of distinguishing between beauty and liking. I think this is a crucial theoretical point which needs to be addressed in the review of the manuscript. I think that in light of this Reviewer advice, the Authors should reconsider whether to modify the title.** The comments that we received concerning this point made us realize that, in fact, our focus was not completely on the spot. In order to fully respond to this one and the related comments, we re-wrote completely the part of the Introduction that assesses our contribution w.r.t. the literature, modified the title, and explained in a clearer way the purpose of our approach. We also improved our discussion.

5. **Please ensure that your manuscript meets PLOS ONE's style requirements, including those for file naming. The PLOS ONE style templates can be found at [. . .].** We believe all requirements were met in our first submission, except the specific one regarding naming of figures. We fixed that now; should any other requirement not being met despite our efforts, please indicate it specifically for us to address it, thank you.

6. **Please provide additional details regarding participant consent. In the ethics statement in the Methods and online submission information, please ensure that you have specified (1) whether consent was informed and (2) what type you obtained (for instance, written or verbal, and if verbal, how it was documented and witnessed). If your study included minors, state whether you obtained consent from parents or guardians. If the need for consent was waived by the ethics committee, please include this information.** We now included all such information in the paper.

7. **4. Please include a caption for figure 1.** Done.

To Reviewer 1

1. [. . .] **Perhaps it could be good to clarify a bit more what the distinction between these two approaches exactly amounts to (perhaps with some clearer examples) and the role (if any) that the distinction plays in the authors' research.** As a matter of fact, we did not want to stress this particular difference so much, as it does not play too much of a role in our treatment. We merely wanted to give a gentle introduction in which the philosophical and the neurophysiological are separated. We modified the text now.

2. **The authors should perhaps clarify a bit more the relevance of the specific studies they mention for their study.** Related work has been entirely re-written now, to ensure that all relevant works are cited. This helped us to focus our contribution in a better way.

3. **Can the authors motivate (in their reply to these comments or in the paper) the exclusion of the data obtained with the other procedure?** Yes. As mentioned, these data are part of a larger project, which includes, as the reviewer has noticed, other source of bio-signals from the subjects. As a matter of fact, after initial pre-processing, we realized that some of these bio-signal do not, in fact, seem to contain relevant information about the problem at hand, and we

have excluded them from the start. In another case, eye-tracking in particular, we do believe that the bio-signal is interesting towards our goal; on the other hand, the methods, computational requirements, and approaches to treat eye-tracking differ completely from those for EEG, which led us to choose among them for the time being. In addition, our methods do allow multi-frame learning, that is, learning from different sources, but only at a prototypical level. In conclusion, our choice to limit ourselves to EEG is justified by too much technical differences between the available signals, which in turn suggests that we shall treat eye-tracking in a separate project. We added a brief justification in the paper as well.

4. **The preprocessing of EEG data seems OK from my non-expert vantage point. Perhaps some slight troubles might come from the prepocessing of the data on liking, which were not normally distributed (see p. 6). It is not entirely clear to me why the authors binned the data the specific way they did, so maybe a few words could be added on that.** We have added a figure that shows the actual distribution. As it can be seen, while it is not normal, it can safely treated as if it was binning-wise, due to its symmetry. In this way, we could avoid more unnatural choices for binning.

5. **I am just a little bit unsure (for lack of expertise) about the authors' use of the "leave-p-out method", particularly regarding their choice to limit themselves to 10 repetitions out of all possible combinations (see p. 11). Maybe in this case too the authors' choice could better justified?** Let us start by recalling that k-fold cross-validation would be the standard approach, here. However, this case is about comparing dataset with very different cardinality; approaching the smallest one in k-fold cross-validation mode would have implied training models with too few instances, so we needed a different approach to ensure comparability. 'Leaving p out' is its most natural generalization, and in order to be as standard as possible, we have set p=10 (typical k-fold cross-validation is used with k set to 10)). Observe, also, that the number of combination of p instances in a set can easily explodes as p grows. We have added a few comments and a bibliographic reference to support these claims.

6. **Some of the results of the experiments with temporal decision trees as reported in the body of the text at p. 12 seems to differ from those reported in the Tables 2 and 3 at the same page. For example, the authors say that when using both and the models extracted on D41 retain an average sensitivity of 78%, but the table suggests instead a value of 66%. Similarly, the authors suggest that the models extracted from D41 using 10 electrodes show a sensitivity of 81%, but the table suggests a more modest 75%. A similar thing happens at p. 13, where the authors suggest that the random forest that has been generated from D41 using 10 electrodes, the bands in the spectrum only, and the best 5 measures shows 72% of accuracy: but the value in the table is instead 68%. I might be misreading the table in some way or misinterpreting the text. But if not, the figures in the text need revision, and the results might be slightly less impressive than what the in-text discussion suggests.** During the writing phase we have produced several versions of the tables, to ensure the best reading experience. In doing so, we have misplaced some correct values, and misread them in the corresponding text. This was by no means intentional or aimed to be a maquillage of the results. We have now corrected the text and the name of the columns in the table.

7. **As for the “Conclusions” section, I would have liked to see the method and results of the study placed more resolutely in the context of the existing literature, particularly the literature mentioned in the “Introduction”. How do the models used by the authors advance neuroaesthetics research? How do they score with respect to other computational models of aesthetic preferences (e.g. those based on various stimulus features)?** We have now improved the discussion section to give an account of how our contribution can be compared with the existing literature; however, being the experimental conditions so different than usual, a comparison is to be taken with care.

8. **How exactly these new methods of knowledge extraction contribute to the stated aim of contributing to “synthesising a theory of beauty”? What are the directions for future research in neuroaesthetics opened up by this study? The authors do say that “our results... show performances that are comparable with the existing literature”, but they do not clarify what existing literature exactly they are thinking about.** On the one side, we have now better compared our results with the literature, in statistical terms. On the other side, after having better defined our purpose, that is, working on a theory of liking, not beauty, we have added several comments that should clarify in which sense this is a step forward.

9. **Language is generally good and adequate to academic standards. There is however a tendency towards wordiness and overly long sentences (see e.g. the second sentence of the “Introduction”), especially in the “Introduction”. There are also a few typos and grammar mistakes scattered throughout the document (see e.g. lines 18, 34, 43, 93, 159, 226, 234).** We fixed several typos; in some cases, such as on line 159 of the first version, there is was no typo to begin with (i.e., the expression $(0, 52]$ is mathematical jargon to indicate the interval ”0, excluded, to 52, included”).

10. **Figures are generally clear, of the right format and resolution, and illustrative. Some figures could benefit from a slightly clearer description (e.g., in Figure 2 it is not immediately clear what is highlighted in what colour; Figure 3 could indicate more clearly which frequency band is represented in which line).** Captions have been improved.

11. **Data sharing in this paper[...].** Please refer to our answer to the editor concerning the same point.

To Reviewer 2

1. **However, two questions arise: 1. The uncovering of the rules depends on the quality of the time series, so why is it assumed to be sufficient with EEG signals alone?** It is true that the uncovering of rules depends on the quality of the series. On the other hand, we do not claim that EEG is enough. We wanted to answer the question of whether EEG carries, or not, information about the sense of liking (as a matter of fact this is, as the reviewer suggests, a better definition). TDT can, in principle, be used on other bio-signals, even at the same time, but this was not the purpose of this paper.

2. **Why should we consider the brain to exhibit rule-based decisions in liking judgment? Perhaps the authors are not interested in uncovering the relationship between neuronal activity and liking, which is critical**

in neuroaesthetics. Perhaps the authors are interested in applying a TDT to a neuroaesthetic case. If so, the quality of the data is critical. Our purpose was and is to find out whether or not 1) the brain exhibits patterns that can be linked to the 'liking' phenomenon, and 2) if TDT is a suitable technique to extract such patterns. The literature seems to indicate that the answer to question 1 may be positive; this means that the purpose of the paper is biased towards question 2. However, as it has been noticed, our experimental conditions are different, and the problem is more difficult. We believe that we have answered positively to both questions, and our purpose cannot be reduced to 'applying a TDT to a neuroaesthetic case'.

3. **While numerous measures are captured, the authors end up using only (dry electrode) EEG signals. This made me wonder whether the gaze pattern, ECG, and EDA could be useful in classifying the self-reported scores. In fact, if the gaze pattern results in a more accurate and sensitive model than the EEG signals alone, it would suggest that the authors are modelling motion artefacts rather than brain signals, which effectively means that the authors are not applying a TDT model to brain signals, but to a combination of convoluted signals. This is not to say that motion is irrelevant to the scores, but it would change the perspective of the paper.** As we have answered to the other reviewer, these data are part of a larger project, which includes other source of bio-signals from the subjects. As a matter of fact, after initial pre-processing, we realized that some of these bio-signal do not, in fact, seem to contain relevant information about the problem at hand, and we have excluded them from the start. In another case, eye-tracking in particular, we do believe that the bio-signal is interesting towards our goal; on the other hand, the methods, computational requirements, and approaches to treat eye-tracking differ completely from those for EEG, which led us to choose among them for the time being. In addition, our methods do allow multi-frame learning, that is, learning from different sources, but only at a prototypical level. In conclusion, our choice to limit ourselves to EEG is justified by too much technical differences between the available signals, which in turn suggests that we shall treat eye-tracking in a separate project. We added a brief justification in the paper as well.

4. **It is unclear whether this paper is dealing with one or both of the following important issues: to quantify the quality of liking, or to give quality to the observed quantities in the model, namely, the rules. If I understood this correctly, the "interpretation" that the authors aim to regain is of the model, not the cortical activity. If so, the quality of the data is immensely important. Needless to say, data quality determines model quality. However, the authors chose to not clean their EEG data.** This is an important point. Our initial statement about not cleaning the data is imprecise. The standard, accepted approach to signal preprocessing in EEG is composed by several steps. We proceeded as in the standard reference literature (see the Preprocessing section), except for the eye-related noise. In order to lower the complexity of the experiment, and considering the nature of the techniques used in the paper, we did not collect eye-related events. We improved the quality of our explanation in the text, now.

5. **Typo on p. 5, line 159. It should be '[0, 52]'.** As we have answered to reviewer 1, the expression $(0, 52]$ is mathematical jargon to indicate the interval "0, excluded, to 52, included". We have explained that in the paper now.

6. **It is unusual to divide brain signals into equally wide bands of frequencies. It makes sense to divide your dataset for computational purposes, but this is at the cost of interpretation. Why would it be useful to model the frequency range of 44 Hz to 48 Hz? In other words, why not simply divide your signal directly into the five wave patterns?** Among the several differences between our approach and the existing ones, our computational method allows us to work with separate bands, arbitrarily wide (or thin). In this way, we do not need to accept the premises that the accepted bands will certainly show all behaviours that are expressed by the EEG, but that for specific patterns (in our case, the liking pattern, assuming that there is one) different frequencies may contribute. Observe that most of the importance of our initial selection work is based on this assumption. So the question should be: why showing the classic bands in the end? The answer is that in this way we give a post-hoc confirmation that, in fact, liking patterns are connected with certain bands, as one could expect, though there are some subtle differences that would have otherwise been overlooked.

7. **Figure 2 and 3: Please add the unit (μV I assume?) and be more concrete about each plot. It's difficult to understand what is what. As they look identical, they are surely not band-pass filtered signals. They should look drastically different from one another if that was the case.** Of course they are not band-pass filtered signals. Figure 2 depicts the first five minutes of recording of the raw signal of an instance. Each one of the subfigures in Figure 3 represents the intensity of voltage varying the time at a given range of frequencies (and, thus, after applying the Furier transformation). They do look similar because such intensities do not vary too much from frequency to frequency; they are not identical, though: first, one can see the average intensity at δ, θ, α is clearly different from that at β, γ, and, second, the first group has clearly less standard deviation than the second. We now improved our captions, as suggested.

8. **Figure 4: Please include what the various shades of blue mean. It should not only be in the text.** We now did.

9. **Typo on p. 9, line 243. Remove the text 'fix.'** We did.

10. **On page 11, lines 319-324: When designed as a classification problem, did you not expect your model to improve its accuracy when the data approaches a binary structure?** Yes. In fact, we only wanted to answer the question 'by how much'. We believe that approaching the problem in this way helps us to understand the intensity that the patterns present. This in turn led us to the observation that the reviewer him/herself found valuable (**I find this observation very valuable. This is an interesting quality observed in the model: your participants suggest that when it comes to liking or disliking a painting, they tend to not have a middle ground.**)

11. **On page 12, lines 329-333: It is very unclear from where these numbers are coming. Using Table 3, I fail to identify the said numbers. In fact, dataset D41 (under beta + gamma) never scores higher than 75 in sensitivity. Are the reported numbers correct?** As we have answered to reviewer 1, during the writing phase we have produced several versions of the tables, to ensure the best reading experience. In doing so, we have misplaced some correct values, and misread them in the corresponding text. This was by no means intentional or aimed to be a maquillage of the results. We have now corrected the text and the name of the columns in the table.

12. **If possible, a few sentences on why/when TDTs are the better choice over HMMs, for instance, would be valuable to the reader. The fact remains that both HMMs and TDTs can be useful for modelling brain signals and can provide some level of interpretability, but the specific strengths and weaknesses of each model may make one more suitable than the other. Why, in your case, is TDT the better approach? I do want to remind the authors that HMMs are particularly useful for modelling systems with a limited number providing insights into the underlying dynamics of the system through the learned model parameters. In giving quality to the quantity (model parameters vs. decision rules), why are decision rules better in your case? These were the thoughts I had while reading your manuscript. A few sentences on this topic would, in my opinion, improve the quality of the paper.** In reference to other comments of this one and the other reviewer, it seems that we had to improve our literature review. We did so, and observed, especially in the Introduction, that it is huge. Having to find a way to 'stay on focus' (this is not a review paper), we have excluded the literature in which experiments and aims were not immediately comparable to ours. HMM have been used to read biosignals, even EEG, but for event detection, movements, epilepsy detection, sleep staging, among other, and not for emotion classification. From a more methodological point of view, HMM can be considered functional methods; they can provide a certain degree interpretability, but it cannot be immediately compared with logical interpretability. Unlike other functional models, HMM focus on the temporal component, and allow one to describe a dynamical process; that is, however, different from extracting explicit logical rules. We added a brief comment in the paper.

13. **It is unclear whether the authors [. . .] unnecessary deep waters.** First of all we recognize that our purpose was to study 'liking', not 'beauty'. We have changed both the title and the claims about our purposes. More in detail, a TDT model gives insights about both quality and quantity; specifically, it is qualitative in the nature of time (before, after, during) and quantitative in the nature of the events (such an electrode register such a spike at such a frequency). Nowhere we claimed that the brain works following a decision tree; a decision tree is merely an intermediate step that serves the purpose of extracting a qualitative/quantitative rule which, in turn, should be interpreted as indication of the underlying brain process, whose validity as the merits and the limits of the statistical value to which is associated.
