# Supplementary material for: Towards an objective theory of subjective liking: A first step in understanding the sense of beauty
Source: PLoS One. 2023 Jun 23;18(6):e0287513. doi: 10.1371/journal.pone.0287513 (PMC10289447; doi:10.1371/journal.pone.0287513)
Supplement: Attachment — Submitted filename: answersV2.pdf [file pone.0287513.s004.pdf]

Towards an objective theory of subjective liking: a first step in understanding the sense of beauty

S. Mazzacane[1], M. Coccagna[1], F. Manzella[2], G. Pagliarini[2], A.V. Sironi[3], A. Gatti[4], E. Caselli[1], G. Sciavicco[2]

## To the Managing Editor

Dear managing editor, thank you for taking the time to handle our paper. As it turns out, reviewer 1 is already happy with the current version. Here, we answer to all questions of reviewer 2.

1. **If the Authors decide not to modify the preprocessing of EEG data as suggested by Reviewer2, they should justify their choice in the manuscript.** We analyzed this question very deeply, and we discussed it among the co-authors. We feel that we are able to justify why we decided to proceed with the current pre-processing, based, on top of other considerations, on recent literature in which similar considerations have been made.

2. **Furthermore, I suggest the Authors to include a wider literature review on neuroaesthetics**. In order to answer the previous point, we widened our literature review, as requested.

3. **Please review your reference list to ensure that it is complete and correct. If you have cited papers that have been retracted, please include the rationale for doing so in the manuscript text, or remove these references and replace them with relevant current references. Any changes to the reference list should be mentioned in the rebuttal letter that accompanies your revised manuscript. If you need to cite a retracted article, indicate the article's retracted status in the References list and also include a citation and full reference for the retraction notice.** We do not believe we have cited any retracted paper; we double-checked our bibliography, just in case.

## To Reviewer 2

1. **Have the authors made all data underlying the findings in their manuscript fully available? "No"** In fact, we had already published the dataset used in this work on Kaggle following PLOS Data policy requirements.

2. **The authors generally answer all my points to a satisfactory level except for the preprocessing point. [. . . ]** This point, indeed, requires a deep analysis. We recognize that we had not discussed this in a satisfactory way in the paper. We have now improved this point. Shortly, there are three arguments relevant here. First, our technique is designed to be used 'online' if necessary. In other words, learning of models, which is offline, is a slow process that requires several days of computation; the resulting model, though, is symbolic [this one of the novelties of our approach], and therefore classification is basically real-time. Now, all pre-processing required, including Fourier transform,

can be realized real-time; yet, the 'missing' pre-processing step (requiring PCA + ICA + ICA inverted + PCA inverted) cannot be realized real-time for computational reasons: every model trained after ICA polishing must be then used only on ICA-polished data [in a sense, the less pre-processing required the more robust is the model]. Second, our entire process starts with a selection phase that identifies the electrodes that are more likely to carry the information of interest. Now, the literature on this point is clear, and indicates which electrodes are more likely to be influenced by eye movements and neck muscle contraction (see, to this end, "Ocular artifacts in EEG and event-related potentials I: Scalp topography" and "Muscle artifacts in multichannel EEG: Characteristics and reduction"). By comparing the positions of the electrodes that have been automatically chosen by our selection and those that are indicated to be possibly under the influence of that type of noise, we see that the intersection is essentially void. In other words, by selecting electrodes specifically for the task, it seems that the learning becomes 'blind' to eye movements and muscle noise, matter-of-factly eliminating the need of selecting the components by-hand. Observe that, more abstractly, if our models were, in fact, affected by noise, this should have emerged in the shuffle post-hoc verification, which we performed. Third, some authors seem to have proceeded in a way very similar to us, that is, they have used machine-learning techniques for neurological tasks from EEGs and did not perform, as a pre-processing step, the eye movement component elimination (see "EEG sub-bands based sleep stages classification using Fourier Synchrosqueezed transform features", 2012, and "Emotion detection using electroencephalography signals and a zero-time windowing-based epoch estimation and relevant electrode identification", 2021). Summarizing: non-ICA-preprocessed symbolic models should be more robust and real-time, the relevant brain areas, in terms of electrodes, should not be affected by eye movements and neck muscle contraction, and other authors seem to have recently followed a similar process.

3. **Line 2: To support your case of distinguishing between 'liking' and 'wanting' in neuroaesthetics, you may consider: 'Chatterjee A. & O. Vartanian. 2014. Neuroaesthetics. Trends Cogn. Sci. 18: 370–375.'** Thank you, we now cited this as you suggest.

4. **Line 3 to 8: There are many "in which"—are you sure you want that structure in your sentences?** We believe that the sentence should be clear enough, in fairness.

5. **Line 13: It is clear that you are familiar with the Weber-Fechner law and surely know that is not an attempt to quantify beauty. It is a law describing the relationship between physical stimulus and perceived change. Your sentence comes across as if it is a law suggested to quantify beauty.** We added a sentence to avoid such a confusion.

6. **Line 20 to 22: To support your case of using 'liking' as an emotion, you may want to consider 'Berridge K. & P. Winkielman. 2003. What is an unconscious emotion? (The case for unconscious "liking"). Cogn. Emot. 17: 181–211.'** Thank you, we now cited this as you suggest.
