# Supplementary material for: Towards an objective theory of subjective liking: A first step in understanding the sense of beauty
Source: PLoS One. 2023 Jun 23;18(6):e0287513. doi: 10.1371/journal.pone.0287513 (PMC10289447; doi:10.1371/journal.pone.0287513)
Supplement: Attachment — Submitted filename: Review of Towards Measuring the Sense of Beauty.docx [file pone.0287513.s001.docx]

Review of **Towards Measuring the Sense of Beauty**

**Short summary.**

The authors' main aim is to understand the link between the amplitudes of voltage at predetermined frequencies on the sensor level of the EEG signals, and the self-report of beauty while standing in front of several paintings. They are motivated by the fact that prior models offer poor interpretability compared to temporal decision trees (TDTs). As a TDT allows extracting specific rules about the signal and reported outcomes, it is motivated as a valuable model with easily interpretable measures.

**Review**

I generally find the symbolic approach to be an underestimated data-driven classification method. As the authors display, it clearly holds great potential in revealing features in the data that may not be picked up by functional approaches. Even more, using more ecologically valid scenarios is exactly the right development for neuroaesthetics being an interesting discipline that has only just begun realizing the importance of context. The authors are certainly on the right track.

There is an increasing trend in applying decision trees to time series, so this is a timely contribution (pun intended). As a TDT allows extracting probabilistic rules, their practical value is evident: if a temporal system is making rule-based decisions, a TDT will surely uncover the rules. However, two questions arise: 1. The uncovering of the rules depends on the quality of the time series, so why is it assumed to be sufficient with EEG signals alone? 2. Why should we consider the brain to exhibit rule-based decisions in liking judgment? Perhaps the authors are not interested in uncovering the relationship between neuronal activity and liking, which is critical in neuroaesthetics. Perhaps the authors are interested in applying a TDT to a neuroaesthetic case. If so, the quality of the data is critical.

While numerous measures are captured, the authors end up using only (dry electrode) EEG signals. This made me wonder whether the gaze pattern, ECG, and EDA could be useful in classifying the self-reported scores. In fact, if the gaze pattern results in a more accurate and sensitive model than the EEG signals alone, it would suggest that the authors are modelling motion artefacts rather than brain signals, which effectively means that the authors are not applying a TDT model to brain signals, but to a combination of convoluted signals. This is not to say that motion is irrelevant to the scores, but it would change the perspective of the paper.

It is unclear whether this paper is dealing with one or both of the following important issues: to quantify the quality of liking, or to give quality to the observed quantities in the model, namely, the rules. If I understood this correctly, the "interpretation" that the authors aim to regain is of the model, not the cortical activity. If so, the quality of the data is immensely important. Needless to say, data quality determines model quality. However, the authors chose to not clean their EEG data.

Perhaps as a next step, the authors could apply strong cleaning pipelines to their EEG dataset (see the work of Klaus Gramann) and see whether that makes the features in the EEG signal even stronger. I suspect this might be the case. If so, it would greatly improve the paper. With that said, I do think the authors are on the right track; both in terms of applying TDT to brain signals and self-reports and regarding the application of real contexts in neuroaesthetics.

**Minor issues**

- Typo on p. 5, line 159. It should be ‘[0, 52]’.
- It is unusual to divide brain signals into equally wide bands of frequencies. It makes sense to divide your dataset for computational purposes, but this is at the cost of interpretation. Why would it be useful to model the frequency range of 44 Hz to 48 Hz? In other words, why not simply divide your signal directly into the five wave patterns?
- Figure 2 and 3: Please add the unit (µV I assume?) and be more concrete about each plot. It's difficult to understand what is what. As they look identical, they are surely not band-pass filtered signals. They should look drastically different from one another if that was the case.
- Figure 4: Please include what the various shades of blue mean. It should not only be in the text.
- Typo on p. 9, line 243. Remove the text ‘fix.’
- On page 11, lines 319-324: When designed as a classification problem, did you not expect your model to improve its accuracy when the data approaches a binary structure?
- On page 12, lines 329-333: It is very unclear from where these numbers are coming. Using Table 3, I fail to identify the said numbers. In fact, dataset *D41* (under *beta + gamma*) never scores higher than 75% in sensitivity. Are the reported numbers correct?
- On page 12, lines 333-335: I find this observation very valuable. This is an interesting quality observed in the model: your participants suggest that when it comes to liking or disliking a painting, they tend to not have a middle ground.
- If possible, a few sentences on why/when TDTs are the better choice over HMMs, for instance, would be valuable to the reader. The fact remains that both HMMs and TDTs can be useful for modelling brain signals and can provide some level of interpretability, but the specific strengths and weaknesses of each model may make one more suitable than the other. Why, in your case, is TDT the better approach? I do want to remind the authors that HMMs are particularly useful for modelling systems with a limited number providing insights into the underlying dynamics of the system through the learned model parameters. In giving quality to the quantity (model parameters vs. decision rules), why are decision rules better in your case? These were the thoughts I had while reading your manuscript. A few sentences on this topic would, in my opinion, improve the quality of the paper.

**Major issues**

I identify 3 major issues:

1. It is unclear whether the authors are aiming to demonstrate how TDTs have revealed an important decision rule based on EEG signals, which is valuable to interpreting beauty (quantifying quality)—or, that TDTs provide quantities in the form of rules that can be interpreted as some quality of beauty (giving quality to quantity). The unclarity revolves around the motivation being ‘interpretability’. It is unclear what exactly is meant to be better interpreted from the approach. While reading through the introduction, I was convinced that this paper is about fitting a model that allows us to better interpret the neurophysiological data relative to the subjective experience of beauty. It was not until page 11 that I realized the aim may be the exact opposite, namely, to give quality to quantity, which in itself is an important (methodological and philosophical) task. It is important to realize that these are two different objectives. Quantifying quality is to demonstrate how, for instance, (spatial/temporal features of) neural signal(s) correlate with self-reports, revealing the measurable structures of beauty, whereas giving quality to quantity (qualitifying quantity?) is to assume that the modelled system (the brain or the subjective valuation system, in this case), truly operates as the structure of the model (a decision tree, in this case), which is quite a claim. I believe I got confused about the aim as the introduction is strongly dominated by neuroaesthetics, which is centred around quantifying quality.
   - If the authors do mean to interpret beauty, it would be more beneficial to introduce what the observed signals from the EEG might mean both neurophysiologically and subjectively. Clearly, the EEG signal is not directly measuring amygdala activity, which the ‘introduction’ discusses, but then what are you classifying, neurophysiologically speaking? By introducing a theory, the interpretation, i.e. quality of quantity, becomes clearer.
   - If the authors do not mean to interpret beauty too, then I do not see why the introduction needs to make bold claims about objective and subjective beauty when the main aim of the paper is not to interpret beauty, strictly speaking. This is effectively putting the paper in unnecessary deep waters.
2. This leads me to another point, namely, what are the authors modelling? Preprocessing is an important stage, particularly for modelling purposes. The important point is that the authors are motivated by the lack of meaningful interpretability of prior work. Neural networks can indeed get incredibly difficult to interpret. However, interpretation of what exactly? They begin by choosing not to clean their EEG data (despite referring to a paper from authors that pioneer the cleaning of EEG data, p. 3, lines 99-102). This is why/where I believe the theory could matter. If this paper is about the application of a TDT to EEG signals, the authors should engage with cleaning the data in the sense that they attempt to dissociate gate-related noise, muscle noise, blinking and other eye-related noise, etc.
   - Surely, the authors can claim that they are focused on computational interpretability, but at the cost of becoming a methodological (proof of concept) paper about applying TDTs to EEG signals and self-reports. The paper should thus be presented as such and not a case about ‘Measuring the Sense of Beauty’.
3. The authors are confusing liking with beauty. There is a wealth of studies demonstrating the difference between beauty and liking judgment. See for instance the works of Oshin Vartanian, Martin Skov, Marcos Nadal, Anjan Chatterjee, and Helmuth Leder. The title and introduction need a rewrite to align with your data dimensions and methodology.
