# Supplementary material for: Towards an objective theory of subjective liking: A first step in understanding the sense of beauty
Source: PLoS One. 2023 Jun 23;18(6):e0287513. doi: 10.1371/journal.pone.0287513 (PMC10289447; doi:10.1371/journal.pone.0287513)
Supplement: Attachment — Submitted filename: Review of Revised Version.docx [file pone.0287513.s003.docx]

Review of **Towards Measuring the Sense of Beauty**

**Review of the revised version**
The authors generally answer all my points to a satisfactory level except for the preprocessing point. My concern is best expressed in the justification made on page 6, lines 220-223. Both eye movement and full-body movement artifacts can vary in frequency, depending on the type of movement. Eye movement is easily picked up by an EEG and, opposite to gait and locomotion, it affects several frequency bands. It is thus, in my opinion, necessary to demonstrate that eye movement and other motion artifacts have been excluded.

- See in particular section “3.2 EEG activity around a saccade” in “Nikolaev A.R., R.N. Meghanathan & C. van Leeuwen. 2016. Combining EEG and eye movement recording in free viewing: Pitfalls and possibilities. Brain Cogn. 107: 55–83.” This paper shows that eye-related activity is mostly expressed in the gamma range.
- See also Figure 7 in “Plöchl M., J. Ossandón & P. König. 2012. Combining EEG and eye tracking: identification, characterization, and correction of eye movement artifacts in electroencephalographic data. Front. Hum. Neurosci. 6:.” This paper also shows that eye-related activity is mostly expressed in the gamma range.
- For the case of micro-saccades (not saccades and free-viewing), which I believe this project will contain many of, see “Engbert R. 2006. Microsaccades: a microcosm for research on oculomotor control, attention, and visual perception. In Visual Perception Martinez-Conde S., Macknik S.L., Martinez L.M., et al., Eds. 177–192. Elsevier.” showing that these occur all the way down to the frequency band of 1-2 Hz.

To be specific, my concern is that the quality of the model may be based on eye-related activity conflated with brain activity. Surely, the quality of the model is also based on neural activity, but my concern is that it is driven by eye-related activity, which is suspiciously expressed in the gamma range too. This concern can be addressed by simply applying an ICA analysis and removing the identified eye-related components. Alternatively, the eye-tracking data can be included and contrasted, however, as the authors suggest, this may be an entirely different project. In addition, one could run two models with separate preprocessing settings and compare the outcomes. This is, of course, beyond the scope of the presented work.

**Minor issues**
There remain just minor issues that I hope the authors would agree to fix to improve the quality of their paper.

- Line 2: To support your case of distinguishing between ‘liking’ and ‘wanting’ in neuroaesthetics, you may consider: ‘Chatterjee A. & O. Vartanian. 2014. Neuroaesthetics. Trends Cogn. Sci. 18: 370–375.’
- Line 3 to 8: There are many “in which”—are you sure you want that structure in your sentences?
- Line 13: It is clear that you are familiar with the Weber-Fechner law and surely know that is not an attempt to quantify beauty. It is a law describing the relationship between physical stimulus and perceived change. Your sentence comes across as if it is a law suggested to quantify beauty.
- Line 20 to 22: To support your case of using ‘liking’ as an emotion, you may want to consider ‘Berridge K. & P. Winkielman. 2003. What is an unconscious emotion? (The case for unconscious “liking”). Cogn. Emot. 17: 181–211.’
